# Quasi-planktonic behavior of foraging top marine predators

**DOI:** 10.1038/srep18063

**Published:** 2015-12-15

**Authors:** Alice Della Penna, Silvia De Monte, Elodie Kestenare, Christophe Guinet, Francesco d’Ovidio

**Affiliations:** 1 Sorbonne Universités, UPMC Univ Paris 06, UMR 7159, LOCEAN-IPSL CNRS/UPMC/IRD/MNHN, F-75005, Paris, France; 2 Univ Paris Diderot Cité, 5 Rue Thomas Mann, 75013 Paris, France; 3 CSIRO-UTAS Quantitative Marine Science Program, IMAS, Private Bag 129, Hobart, Tasmania 7001, Australia; 4 Ecole Normale Supérieure, Institut de Biologie de l’ENS (IBENS), UMR CNRS 8197 and INSERM U1024, 46 rue d’Ulm, F-75005 Paris, France; 5 Laboratoire d’Etudes en Géophysique et Océanographie Spatiales (LEGOS), Université de Toulouse III (OMP) and IRD, Toulouse, France; 6 Centre d’Etudes Biologiques de Chizé, 79360 Villiers-en-Bois, France

## Abstract

Monitoring marine top predators is fundamental for assessing the health and functioning of open ocean ecosystems. Although recently tracking observations have substantially increased, factors determining the horizontal exploration of the ocean by marine predators are still largely unknown, especially at the scale of behavioral switches (1–100 km, days-weeks). It is commonly assumed that the influence of water movement can be neglected for animals capable of swimming faster than the current. Here, we challenge this assumption by combining the use of biologging (GPS and accelerometry), satellite altimetry and *in-situ* oceanographic data (ADCP and drifting buoys) to investigate the effect of the mesoscale ocean dynamics on a marine predator, the southern elephant seal. A Lagrangian approach reveals that trajectories of elephant seals are characterized by quasi-planktonic bouts where the animals are horizontally drifting. These bouts correspond to periods of increased foraging effort, indicating that in the quasi-planktonic conditions energy is allocated to diving and chasing, rather than in horizontal search of favourable grounds. These results suggest that mesoscale features like eddies and fronts may act as a focal points for trophic interactions not only by bottom-up modulation of nutrient injection, but also by directly entraining horizontal displacements of the upper trophic levels.

Marine top predators play a key role in maintaining the health of open ocean ecosystems and their monitoring is fundamental for assessing the quality of the marine environment, in particular in the wake of a changing climate^1^,^2^. Over the last two decades marine top predators have been the subject of numerous tracking programs^3^,^4^ aimed at identifying key habitats, e.g. foraging and breeding grounds^5^,^6^,^7^,^8^,^9^,^10^,^11^, studying their relationship with oceanographic features^12^,^13^,^14^,^15^,^16^,^17^,^18^,^19^,^20^, investigating their navigation capabilities^21^,^22^ and gathering information about their biotic and abiotic environment, notably on remote oceanic regions^23^,^24^,^25^,^26^. The temporal and spatial resolution of these studies is rapidly improving, enabling the observation of not only large scale migrations, but also the fine scale (~1 *km*) features of foraging trips. This increasing spatiotemporal resolution and the use of accelerometers makes it now possible for the first time to investigate the behaviors that underpin the observed patterns of displacement, and to relate these patterns to the physical properties of the turbulent environment that marine predators experience.

Large predatory fish, marine mammals and swimming seabirds are classified as *nekton* - free-swimming animals - because they are able to swim at a speed that is several times larger than the strongest currents of the open ocean. In contrast, *plankton* - literally meaning “wanderer” or “drifter” - refers to organisms that are passively transported, typically because they have no autonomous capacity of motion, or this is too weak to overcome transport. Consistently with such a view, the mechanical effect of currents on the trajectories of marine predators is often neglected and the classification of their behaviors is typically performed by borrowing approaches from terrestrial ecology, where animal search for food occurs on a faster timescale than the temporal variability of the landscape.

However, both the “nekton” and “plankton” labels are used in a qualitative sense, as most drifting organisms have some propulsion capabilities, and the currents affect any free swimming organism by shifting its frame of reference. Although the swimming capabilities of planktonic organisms have received quite a lot of attention^27^,^28^,^29^,^30^, only few studies have tackled explicitly the question of the extent to which ocean currents can determine the trajectories of large marine animals and have concluded that this effect can be generally neglected. Indeed, the fact that nektonic animals are able to overcome oceanic currents does not imply that their movement is not directly influenced. To our knowledge, horizontal currents have only been shown to offset the trajectories of sea-turtles, which not surprisingly are among the slowest nekton^31^,^32^,^33^. Similarly, Lea *et al.*^34^ identified a relationship between fur seal pups swimming speed and wind speeds during their initial dispersal in extreme wind events. Instead, the need for correcting tracked trajectories of fast swimmers, such as elephant seals or whale sharks, has been ruled out by comparison with satellite altimetry^35^ and synthetic water parcels advected in numerical models^36^.

Interestingly however, Campagna *et al.*^37^ present a striking example of elephant seals whose long trajectories (>500 *km*) closely resemble that of a drifter released almost simultaneously in their proximity. Is this example merely anecdotal or should the common assumption be revised? This question is important in marine ecology because the assumption that a swimming behavior is not directly affected by the ocean currents stands at the core of the analysis of search behavior of fast swimming animals - the majority of tagged species. This assumption has enabled this field of research to borrow techniques from terrestrial ecology, and notably the classification of animal behavior based on the sinuosity of trajectories for the identification of foraging grounds. On the other hand, if currents had sizable effects on the displacement of a marine predator, then ocean circulation features - like eddies and fronts - may play an important role in structuring the ecosystem not only bottom-up, but at multiple levels of the food chain. Besides, being hotspots of primary production, they would entrain top marine consumers, and thus concentrate in the same locations different organisms and their trophic interactions.

Here, we aim at assessing the impact of ocean currents on one of the fastest marine predators for which high-resolution tracking is available: the southern elephant seal. Using a novel biologger, we analyse simultaneous tracking (GPS - Global Positioning System) and behavioral data (accelerometry) from female southern elephant seals, *Mirounga leonina*, from the Kerguelen Islands (Indian Sector of the Southern Ocean, see Fig. 1). Southern elephant seals are a model species to address our research aims. During their long-range foraging trips, these animals encounter different oceanic regimes, and in particular highly energetic features emerging between the Polar and the Sub-Antarctic fronts. Their size allows them to carry with minimal disturbance large bio-loggers with long-lasting batteries and multiple detectors, among which accelerometers that can provide a direct estimation of capture attempts independently of the trajectory analysis. The Kerguelen sub-population, the second largest in the world, is the subject of an ongoing decadal study in terms of demography^38^,^39^ and animal tracking^14^,^40^,^41^.

Our study focuses on the scale at which switches in the patterns of foraging behavior of marine predators are observed (~10 *km*). In terms of ocean physics, this is the (sub-) mesoscale. This scale is also referred to as *ocean weather* because of the presence of eddies and frontal systems^42^,^43^,^44^ similar to meteorological systems. Such dynamical structures shape the distribution of chemico-physical tracers such as Sea Surface Temperature^45^, biotic fields like Chlorophyll concentration^46^,^47^,^48^,^49^,^50^ and community composition^51^. Furthermore, mesoscale turbulence is a major determinant of the distribution of consumers as zooplankton^52^ and micronekton^53^. Top predators such as whales^13^, squid^54^, king penguins^16^, sea-turtles^55^, frigatebirds^15^,^20^, elephant seals^35^, fur seals^56^,^57^ and albatrosses^58^ have been observed to co-localise with mesoscale structures but how much these animals actively track these features and how much they are entrained by them is part of the open question which we address here. In this study we find that, in contrast to what is often assumed, all the analyzed trajectories are characterized by bouts that are largely dominated by the currents advection and we develop a Lagrangian method to quantify the contribution of horizontal oceanic currents to an animal trajectory.

## Method

**Figure 1:**
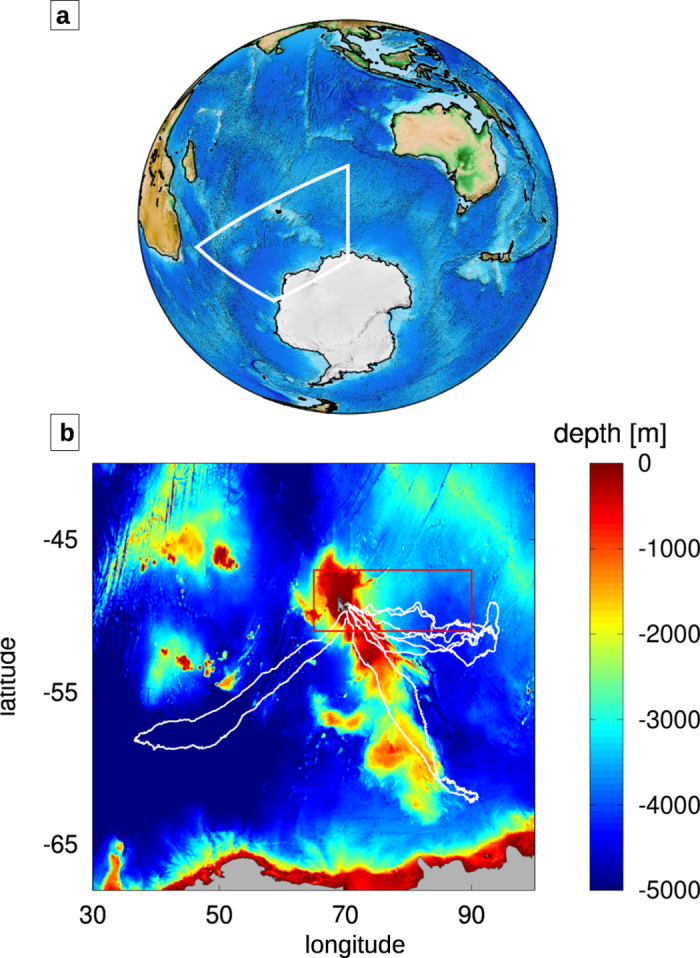
The data employed in this study refer to the Kerguelen region (white polygon), in the Indian Sector of the Southern Ocean (a). (**b**) The trajectories of the tagged elephant seals (white) overlapped with the bathymetry of the region. The red rectangle identifies the sub-region containing the trajectories of the drifting floats released during the KEOPS 2 campaign. Bathymetric data from ETOPO2 Global 2-Minute Gridded Elevation Data Volume E1 [U.S. Department of Commerce, National Oceanic and Atmospheric Administration, National Geophysical Data Center, 2001. 2-minute Gridded Global Relief Data (ETOPO2), access:8/30/2001].

The elephant seals tracking dataset employed in this study consists of five post- breeding foraging trips collected between October 2010 and January 2012 (see Fig. 1). The elephant seals were tagged with GPS transmitters with a space-time resolution of 50 m and 20 minutes respectively. All animals in this study were cared for in accordance with the IPEV ethical and Polar Environment Committees guidelines. The experimental bio-logging protocol was approved by the IPEV ethical and Polar Environment Committees. A total of more than 20000 km and 300 days data were recorded, from single trips of 72 to 85 days in duration. The tracking data were filtered by removing the locations that would have implied seals velocities larger than 2.8 *m*/*s* according to the algorithm described in References^5^,^14^. Seal velocities larger than this threshold are unrealistic and likely to be due to GPS errors. Individual seals were also equipped with accelerometers to detect rapid head movements that characterize prey capture attempts. Accelerometers allow the identification of prey captures with an accuracy of more than 80%^5^,^59^,^60^,^61^. However, due to limited battery power, for the three longest trajectories we were only able to measure the rate of prey capture for the first part of the foraging trip (about half of the round trip, or ~2000 km). Both GPS transmitters and accelerometers have been observed not to interfere with marine mammal behavior^60^,^62^,^63^. Following References^31^,^32^, we define *tracking velocity* the velocity estimated by differentiating in time the GPS positions and *heading velocity* the tracking velocity minus the estimated velocity of the ocean currents. In practice, the tracking velocity corresponds to the speed of the animal in a fixed frame of reference, and the heading velocity to the component relative to the moving water parcel the animal belongs to.

Geostrophic currents were quantified through an altimetry multi-satellite global product (Delayed Time Maps of Absolute Dynamic Heights (DT-MADT)) developed by CNES/CLS Aviso (http://www.aviso.oceanobs.com). This product has temporal and spatial resolution of respectively 1 week and 1/3° 64. Regional versions of the product, one of which is corrected with wind-induced Ekman component at 15 m (the depth of the SVP - Surface Velocity Program- drifters’ drogue), have been also used. A comparison between the Lagrangian diagnostic introduced in this study -the Quasi-Planktonicity Index, see later- computed using different remote sensing products is shown in the Supplementary Information. Although the findings of this study do not depend on the choice of specific altimetric products, the presented results are obtained by using geostrophic products, that better refer to the typical diving depths of elephant seals, as detailed in the discussion.

The altimetry-derived velocity field was used to evaluate the heading velocity^32^ and to compute the synthetic trajectories of virtual drifters. Simulated trajectories have been obtained by integrating the velocity field through a 4th order Runge-Kutta algorithm and allowed to compute two Lagrangian diagnostics: the Quasi-Planktonicity Index (QPI) -which we introduce in this paper- and the finite-size Lyapunov exponent (FSLE). This exponent is obtained by measuring the backward-in-time divergence of initially nearby particles and it is commonly used as an indicator of frontal activity and stirring intensity. Indeed, highest dominant FSLE values are associated to formerly distant water masses, whose confluence creates a transport front^65^,^66^. Fronts identified as maxima (ridges) of FSLEs have a convergent dynamics transverse to them, so that passive particles - like plankton or drifting buoys - in their neighbourhood are attracted to the front and then advected along it. Following Reference 67 we refer to these fronts as attractive Lagrangian Coherent Structures.

The Lagrangian features of elephant seal trajectories were compared with those of 47 WOCE-SVP drifters (GDP – http://www.aoml.noaa.gov/phod/dac/index.php) released during the multidisciplinary cruise KEOPS 2 (November 2011). The cruise and the release of the drifters took place in a sector of the region explored by the elephant seals trajectories and during the same season when trajectories were recorded^68^. Some examples of drifters trajectories are shown in the Supplementary Information (Fig. 1).

Because elephant seals are diving predators^69^, we used more than 20 casts (see Supplementary Information for more details) of two RD Instrument 300 kHz lowered acoustic Doppler current profilers (LADCP, also from the KEOPS2 cruise) to relate the horizontal currents integrated over the average diving depth (500 m,^61^) with those of the upper layer (here approximated at 50 m) that we infer from altimetry.

Multivariate statistical analyses were performed using linear mixed effect models (“lmer” function in the R package^70^) to relate the number of prey capture attempts -response variable (fitted with a Poisson distribution) -, to the standardised (centered and scaled) frontal activity (FSLE) and QPI - explanatory variables. Individual seal identity was included as a random effect to account for the individual variability.

## Results

**Figure 2:**
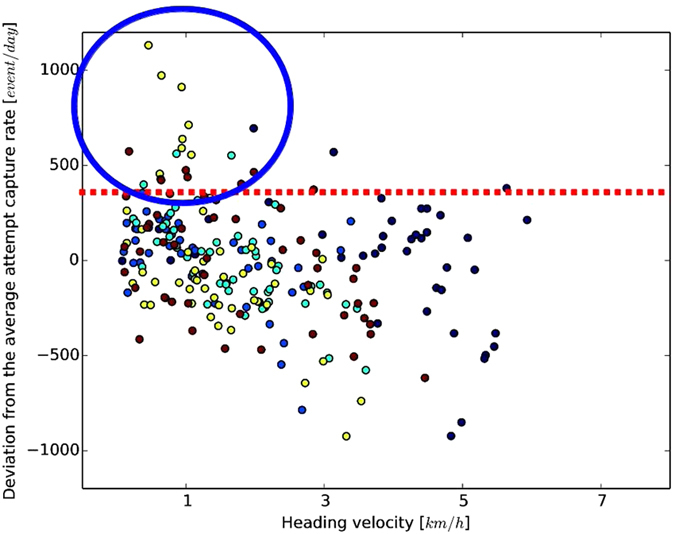
Deviations from the individually-averaged attempt capture rate for different values of heading velocities. Different colors correspond to different individuals. 85% of attempt capture rate of intensive foraging (deviations larger than 300 event/day, above the red dashed line) correspond to of heading velocity below 2 *km*/*h* (blue circle).

The comparison between the heading velocity of elephant seals and the accelerometry data (see Fig. 2) along trajectories shows that when foraging more intensively (with attempt capture rate deviation from the average larger than its standard deviation), the tracking velocity of the elephant seals is close to the geostrophic current measured in the same location (i.e. the heading velocity is small). As displayed in Fig. 2, 85% of the intensive foraging locations correspond to heading velocities below 2 *km*/*h* and a significant (*p* − *value* < 0.01) negative correlation of −0.34 suggest a relation between heading velocity and foraging behavior.

Do the low values of heading velocity imply that elephant seals in intensive foraging activity are “locked” to a specific water parcel and horizontally transported within?

By only considering heading velocities, it is not possible to answer this question as the small values that the heading velocities have in these cases could lead to a large trajectory difference when integrated in time.

Therefore, in order to quantitatively associate horizontal passive movement to a predator’s trajectory, we compare the animal’s trajectory to that of real buoys and of virtual particles purely displaced by advection, obtained by integrating altimetry-derived currents in time. To this aim, we define a new Lagrangian diagnostic: the Quasi-Planktonicity Index (QPI). For each day along an elephant seals’ trajectory, we initialize a synthetic passive tracer in a disk centered around the current animal’s location and we simulate the motion of the particles contained within the disk forward in time for 4 days. We define as *shadow trajectory* the synthetic trajectory closest to the path the elephant seal actually takes in the following 4 days (see SI for the details about the definition of the distance and its computation). The value of the QPI is the mean distance between the observed and the shadow trajectories. In other words, the QPI measures the offset over a four day period between the animal trajectory and the trajectory of a virtual drifter released next to it at the starting position over a four day period.

**Figure 3:**
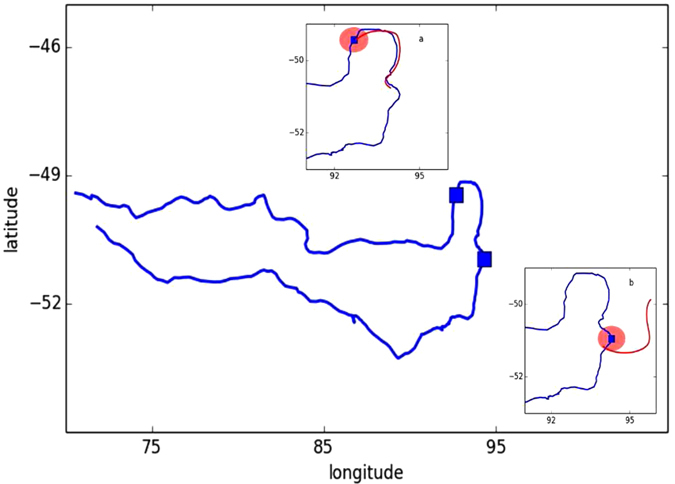
Two examples of computation of the QPI (Quasi-Planktonicity Index) along the same individual’s trajectory (blue line). Disks of simulated trajectories (red disks) are initialised around two locations along the trajectory of the elephant seal (blue squares). The simulated trajectories are the result of the only effect of the currents and the one that minimizes the distance from the elephant seal’s one is used to compute the QPI. The QPI corresponds to the average distance between this trajectory (red lines) and the elephant seal’s. In case (**a**) (*QPI* = 7.8 *km*) the two trajectories resemble each other whereas in case (**b**) (*QPI* = 46.1 *km*) they diverge.

Figure 3 displays two examples of the computation of the QPI along two different sectors of the same trajectory (in blue). The red patches represent the disk of initialized trajectories and their size takes into account of the uncertainty on the initial condition due to the error induced by altimetry resolution. The shadows trajectories are represented in red: in case a), corresponding to a QPI = 7.8 km, the shadow trajectory closely resembles that of elephant seal, whereas in case b), referring to a QPI = 46.1 km, the trajectory of the elephant seal appears strongly uncorrelated with that of the simulated tracer.

**Figure 4:**
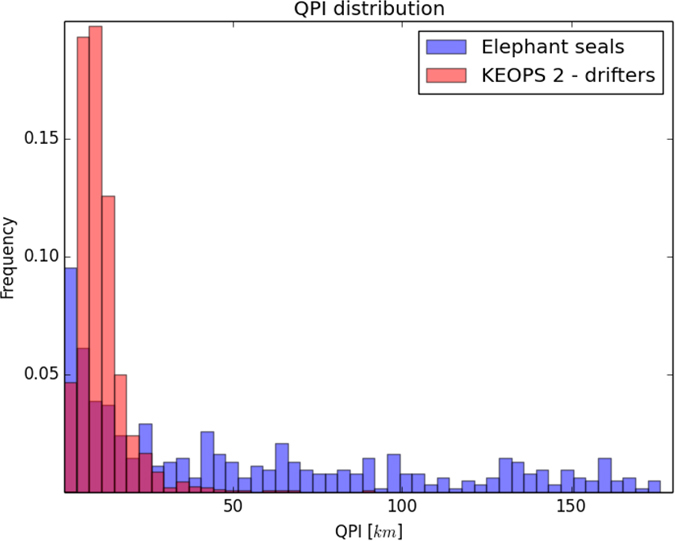
Normalized distribution of values of QPI for buoyant drifters (red) and elephant seals (blue). The extent of the drifters’ distribution suggests that values of QPI below the 20 km threshold refer to bouts of elephant seals’ trajectories where they are considerably affected by the horizontal currents.

Cases such as that illustrated in Fig. 3a) account for on average more than 30% of the time along a foraging trajectory. The values of the QPI in these bouts are compatible with the trajectory being generated by passive advection. This is confirmed when we compare them to the values obtained by applying the same diagnostic to SVP (real) drifter trajectories. Figure 4 shows the distribution of the QPI computed for elephant seals and for 47 SVP drifters. The considerable overlap between the two distributions suggests that values of the QPI below the 20 *km* threshold refer to bouts of elephant seals’ trajectories where the animals display horizontally passive, quasi-planktonic behavior. This result does not change quantitatively if different altimetry products are used, as detailed in the Supplementary Information, and even when the movement of SVP drifters are corrected for the wind-induced Ekman component, that affect the movement of SVP drifters.

When the trajectories are strongly affected by the horizontal dynamics, physical forcing acts as a major driver in the exploration of the horizontal space. As a consequence, animals are expected to be found more often on attractive transport structures induced by horizontal stirring. Attractive Lagrangian Coherent Structures (LCSs) can be identified by remote sensing as ridges of Finite-Size Lyapunov Exponent (FSLE)^66^. A multivariate analysis through a linear mixed effects model (see Methods) reveals highly significant (*p* − *values* < 0.001) correlations between FSLE, the QPI and the rate of attempted prey capture (see Table SI1). These results indicate that on transport fronts the tracked elephant seals are passively advected to a higher degree (they have smaller QPI) and forage more intensively.

**Figure 5:**
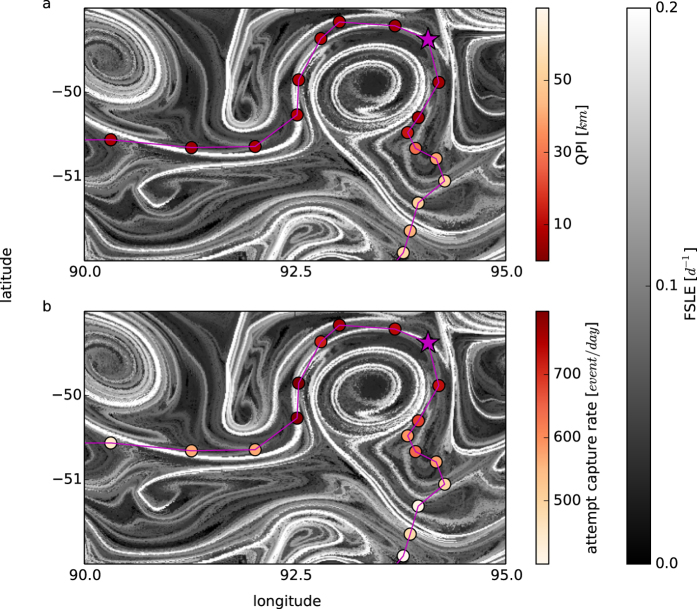
Fronts, identified as FSLE ridges (gray scale background image in both (a) and (b), computed the day corresponding to the location marked as a purple star(02/12/2011)) correspond on average to lower QPI values (a) and higher attempt capture rates (b). Note that the colorscales are reversed to better highlight that lower QPI correspond to higher attempt capture rates. In most cases locations with low QPI correspond to high capture rate. However, cases excepting this trend (in this example the locations of longitude between 90–92.5°) suggest that elephant seals could present a quasi-planktonic behaviour in response to physical clue usually, but not always, associated to rich foraging grounds.

Figure 5 shows a typical example of this correlation. The gray-scale image in the background refers to the FSLE (the lighter the color, the stronger the transport-induced front). The points along the elephant seal’s trajectory are colored according to the QPI (Fig. 5a) and the attempt capture rate (Fig. 5b). As the capture rate increases, the QPI decreases (note that the color scales are reversed), meaning that the trajectory is more affected by the currents. This situation occurs in regions of high FSLE, indicating transport fronts, whereas outside of fronts there are no recognizable patterns in either of the two behavioral diagnostics.

These results are obtained by assuming that the geostrophic currents are representative of the ones experienced by diving elephant seals. This assumption is checked by using vertical profiles of horizontal velocities from ADCP: the correlation between the zonal and meridional components of the velocities at 50 m depth and the integral between 50 m and 500 m is significant in both cases with values over 0.7 (r = 0.7 and r = 0.9 for the zonal and the meridional components of the velocity field – *p* − *values* < 0.01), indicating that the surface currents provide reliable information on the horizontal advective drift experienced by the animals during their diving.

## Discussion

The results of this study challenge the common assumption that fast swimming predators have horizontal displacements that are substantially independent of surface currents. Moreover, the distortion of the trajectory caused by horizontal transport appears to occur prominently where foraging is most intense, stressing the importance of taking water movement into account when analyzing animal displacements at the scale of tens of kms. Determining the correct repartition of efforts between active displacement and passive plankton-like behavior is a central requirement for the application to marine mammals of general frameworks such as Optimal Foraging Theory^71^. Such a theory, based on the hypothesis that animals invest a limited amount of energy between foraging-inefficient displacement and targeted local search for food, suggests that the optimal trajectory is composed by an alternation of long exploratory bouts and of clusters of localized movement known as area-restricted search (ARS). If a searcher’s movement is embedded in a flowing medium, however, its trajectory is deformed by the currents. Moreover, such a deformation is not uniform, since it occurs to a different extent, depending on the animal’s propulsion relative to the surrounding water. Instead of being characterized by short and localized displacements, hence, the intensive search in the vertical direction produces horizontal trajectories that are closer to the flow-induced movement of the animal’s frame of reference, and can therefore being considerably stretched out. The Quasi-Planktonicity Index introduced in our study provides a criterion to measure the degree to which the trajectory of a tracked animal is the outcome of advection by the physical flow. The corresponding partition of elephant seals foraging trips supports the conclusion that would be drawn by optimal foraging theory: animals feed more intensively when their horizontal displacements are more passive.

Our results suggest that ARS algorithms used to detect intensive foraging areas of elephant seals may be misleading in the ocean, if the trajectories are not corrected for the effect of the currents. Indeed, in contrast to the terrestrial environment, ARS in the open ocean produce displacements that are localised in the reference frame of the water parcel which contains the animal, hence trajectories that are shadowed by passive drifters. Especially in energetic regions, like frontal systems, quasi-planktonic horizontal displacements may be comparable in length to the bouts resulting from active propulsion. Correcting the trajectories for the effect of the currents is expected to improve the sensitivity in detecting and classifying behaviours, and in particular those related to foraging activity. In this regard, we note that recent work by Cotté *et al.*^72^ highlights a strong association between transport fronts and the Kerguelen elephant seals’ displacements, but surprisingly not with intensive foraging locations. A possible reason may be that the state space model approach used in that work was applied to the absolute displacement of the animals and not the one corrected for the effect of currents.

The partitioning of elephant seals foraging trips into quasi-planktonic bouts and active horizontal displacements supports optimal foraging theory: the possibility of exploring new foraging grounds is traded off for intensive foraging, which entails focusing on the local resources. This horizontal pattern is consistent with elephant seal diving behavior. When foraging intensively, elephant seals generally increase their diving angles and both the horizontal and vertical sinuosity of the local displacements^5^. As a consequence, when diving they reduce their active horizontal movements (Lebras,Y., personal communication), so that their change results in a horizontal displacement movement that is largely determined by the currents. Note also that because of the increased diving effort in foraging regions, the “quasi-planktonic” horizontal bouts do not necessarily corresponds to periods of reduced energy consumption. Energy is invested more in short- range activity (deep diving and hunting) rather than in large-scale horizontal displacement (commuting).

We choose to use the term “quasi-planktonic” to refer to cases where a nektonic animal’s behavior, in our case intensive foraging, results in an increased vertical movement and a reduced horizontal displacements. This behavioral change makes the horizontal trajectory largely affected by oceanic currents.

In this study, we assume that the velocity field of geostrophic currents inferred from altimetry accounts for the horizontal displacement of the water surrounding the elephant seals. If this is probably a good assumption close to the surface, it may only explain part of the animals’ behaviour, since elephant seals however move considerably in the vertical direction as well. They dive to an average depth of 300–500 m (and up to 2000 m)^61^ and spend more than 60% of their lifetime below 100 m depths^73^. If geostrophic velocities are considered to be representative of the mixed layer^74^, their ability to quantify horizontal displacements at depth is not the same in different regions of the ocean. In the Southern Ocean, where the mixed layer is considerably deep (~100 *m*^75^), the vertical distribution of horizontal velocities suggests that geostrophic velocity can reliably represent that of the whole water column^76^,^77^. We checked that this was the case in the region of our study by analyzing Acoustic Doppler Current Profiler (ADCP) velocities from the KEOPS 2 campaign and the results confirmed that the zonal and meridional components of the horizontal velocities at 50 m depth are significantly related to the integral between 50 m and 500 m.

The “quasi-planktonic” nature of intensive foraging bouts implies that transport structures can entrain animal trajectories. This observation agrees with the increasing number of studies on tracked marine predators, showing that they tend to co-occur with thermal and transport fronts^78^. The common explanation for the localization of top predators over fronts is the bottom-up structuring effect of these transport features due to the local enrichment in nutrients entailed by vertical sub-mesoscale circulation^79^. The consequent boost in the biomass of lower trophic levels is believed to attract free-swimming predators over fronts. Our results indicate that a second, top-down mechanism may also exist, by which frontal structures directly entrain the trajectories of actively foraging predators. Passive advection towards attractive frontal regions may cause an increased localization of predators in those areas, in spite of the relatively small portion of the ocean surface they occupy. It is still an open question whether this mechanism is sufficient to ensure an efficient identification of putative nutrient-rich spots, or if the observed distributions also require the guidance of a cue. A question however remains, on the existence of a cue able to initially guide predators towards the putative nutrient-rich spots, that represent a minority of the ocean extension. Some species of seabirds have been observed to respond to chemical cues of compound dimethyl sulfide (DMS), that accumulates in the air above productive ocean areas^80^,^81^, but relatively little is known about potential physical or chemical cues followed by swimming predators. An alternative hypothesis is that seals moving within a global foraging area modify their behavior according to prey density, which is influenced by (sub-)mesoscale oceanographic structures. With our data it is not possible to address directly this question. However, we observed cases in which elephant seals exhibit quasi-planktonic behaviors without foraging intensively, suggesting that they may be not reacting to the density prey as a cue.

## Conclusions

The term *planktonic* has been traditionally reserved to the lower levels of the trophic chain. However, we have shown that top predators, in spite of being capable of large scale active swimming, can also display a (horizontal) planktonic behavior, that we have called quasi-planktonic. This behavior is associated with intensive foraging, where elephant seals displace mostly in the vertical direction, so that the horizontal displacements follow their moving frame of reference. The entrainment by currents of nekton, as well as of plankton, suggests a mechanism which focuses trophic interactions on physical features which have an attractive dynamics transverse to them, - like eddies and fronts – in alternative (or in addition) to bottom up effects expected by nutrients injections and concentration^79^,^82^,^83^,^84^.

Understanding how the behavior of individual predators is modulated by structures that vary on the spatiotemporal scale of tens of kilometers and of days - the (sub) mesoscale - is an essential step in linking marine predators ethology to conservation ecology, and lies at the heart of predicting large-scale patterns of displacement and the response of marine predators to climate change^85^,^86^,^87^,^88^.

## Additional Information

**How to cite this article**: Della Penna, A. *et al.* Quasi-planktonic behavior of foraging top marine predators. *Sci. Rep.* **5**, 18063; doi: 10.1038/srep18063 (2015).

## Supplementary Material

Supplementary Information
