# Supplementary material for: Quasi-planktonic behavior of foraging top marine predators
Source: Sci Rep. 2015 Dec 15;5:18063. doi: 10.1038/srep18063 (PMC4678296; doi:10.1038/srep18063)
Supplement: Supplementary Information [file srep18063-s1.pdf]

Quasi-planktonic behavior of foraging top marine predators

Alice Della Penna[*,1,2,3], Silvia De Monte[4], Elodie Kestenare[5], Christophe Guinet[6], and Francesco d'Ovidio[1]

[*]alice.della-penna@locean-ipsl.upmc.fr
[1]Sorbonne Universités, UPMC Univ Paris 06, UMR 7159, LOCEAN-IPSL, F-75005, Paris, France
[2]Univ Paris Diderot Cité
[3]CSIRO-UTAS Quantitative Marine Science Program, IMAS, Private Bag 129, Hobart, Tasmania 7001, Australia
[4]École Normale Supérieure, UMR 7625 Ecologie et Evolution , Paris, France
[5]Laboratoire d'Etudes en Géophysique et Océanographie Spatiales (LEGOS), Université de Toulouse III (OMP) and IRD, Toulouse, France
[6]Centre d'Etudes Biologiques de Chizé,79360 Villiers-en-Bois,France

Supplementary information

Details about the Quasi-Planktonicity Index algorithm

The QPI compares a section of a seal's trajectory with the movement of a numerical passive tracer that is determined only by the horizontal currents. In the ideal case of a perfectly determined velocity field, if a trajectory is purely passive, the numerical passive tracer should perfectly match the real trajectories. However, in reality there are two sources of error that have to be dealt with:

- **an uncertainty on the initial condition of the velocity field:** the spatial resolution of altimetry is considered to be comparable to its grid spacing, i.e., 1/3°, hence when we initialize a numerical drifter we may actually initialize it with a mismatch of 1/3° in respect to the velocity field,

- **an underestimation of the horizontal velocities:** altimetry observations are taken along satellite tracks and are then interpolated together

for providing a gridded product. The interpolation procedure smooths the signal and may underestimate real velocities, resulting in our case into a *delay* of the simulated trajectories which lag behind real ones and in turn, into a spurious mismatch during the comparison.

- **ageostrophic components:** by definition, ageostrophic components of the velocity fields do not appear as a signal on the Sea Surface Height and therefore cannot be observed by satellite altimetry.

The algorithm to compute the QPI aims at mitigating the effect of these sources of error. To compute QPI, we perform the following steps:

1. sample the elephant seal's trajectory $\mathbf{X}(\mathbf{t}) = (X(t), Y(t))$ with a 6-hours frequency.

2. for each day t_0 we initialize around the location $\mathbf{X}(t_0)$ a set of j initial conditions $\mathbf{x_j}(t_0) = (x_j(t_0), y_j(t_0))$. They represent the initial conditions of a set of synthetic trajectories $D_r(\mathbf{X}(t_0)) = \{\mathbf{x}(t) : |\mathbf{x}(t_0) - \mathbf{X}(t_0)| < r\}$ where r indicates the radius of the disk.

3. advect the initial conditions for a time $t_{max} = N + t_{buffer}$.

4. for each elephant seal's locations between t_0 and t_0+N: $\mathbf{X}(t_0), \mathbf{X}(t_1)...\mathbf{X}(t_N)$ compute the *pseudo-distance* (from now on distance):

$$\Delta(\mathbf{X}(t_0), \mathbf{x_j}; N) = \min_{i \in [0, t_{max}]} (dist(\mathbf{X}(t_0), \mathbf{x}(t_i)) \tag{1}$$

   where *dist* refers to en Eulerian distance computed on the non-regular latitude-longitude grid.

5. compute the QPI as the mean distance between the closest simulated trajectory (shadow trajectory) and the real one

$$QPI(\mathbf{X}(t_0); N, r) = \frac{1}{N} \min_{j \in D} \left(\sum_{i=t_0}^{t_0+N} \Delta(\mathbf{X}(t_i), \mathbf{x_j}) \right). \tag{2}$$

This algorithm limits the effect of the uncertainty on the initial location of the altimetric velocity in respect to the location of the real trajectory (that for the case of elephant seals and SVP drifters we consider with no error, given the high resolution of GPS tracks), by advecting an ensemble of numerical trajectories whose radius r is chosen in relation to the resolution of the altimetry data: in this study we used $r = 0.3°$.

To compensate for a lag in the simulated trajectory we introduce the pseudo-distance defined in step 4 instead of an Eulerian step-by-step distance. Indeed, even if a simulated and a measured trajectory are very close, if the velocity field is underestimated, the Eulerian distance would increase and we would not identify a low value of the diagnostic. Therefore, we advect the simulated trajectory for a t_{max} that is not just equal to the number of steps we use for

Variables	Estimate	Error	z-value	p-value
QPI	-0.258	0.004	-72.50	$< 2 \cdot 10^{-16}$
FSLE	0.015	0.003	5.46	$4.71 \cdot 10^{-8}$

Table 1: **Linear Mixed Effect Model relating** $attempt\ capture \sim QPI + FSLE + (1|ID)$. The variability between individuals is taken into account by considering the ID as a random effect. Both explanatory variables are significantly related to the attempt capture rate, yet the QPI appear to have a stronger effect.

the comparison (N), but we introduce a buffer (in this study $buffer = 4\ days$), so that we make sure that we are compensating for all the effects of the delay. We then ensure that for each position of the real trajectory, the distance is computed with the closest point of the simulated trajectory, and not the point that corresponds to the same instant.

Finally, we addressed the presence of possible ageostrophic components by validating the QPI (i.e., computing it for SVP drifters) also adding to satellite-derived currents the Ekman components derived by wind re-analysis. Figure SI 4 displays the distributions of the QPI computed for SVP drifters using different altimetry products. Even if by using a regional Ekman-corrected altimetry the simulated trajectories have Lagrangian properties that are more similar to the SVP ones, as detailed in Ref [1], the changes in the QPI distribution does not change qualitatively the result.

When computing this algorithm, there are few parameters that can be tuned. If r is constrained by the resolution of the velocity field, the choice of N is relatively flexible and it is related to the scale of the patterns we want to identify. As in this study we are interested in labelling bouts of trajectories with a resolution high enough to distinguish behavioral switches between extensive and intensive foraging (typically of of few days) we used a value of $N = 4\ days = 16\ steps$. The QPI can be computed with different frequencies: in this study we sampled the trajectories every 6 hours, but when comparing the QPI with the attempt capture rate, we used a daily resolution to integrate for the effects of the day-night cycle of the attempt capture rate.

References

[1] d'Ovidio, F. *et al.* The biogeochemical structuring role of horizontal stirring: Lagrangian perspectives on iron delivery downstream of the kerguelen plateau. *Biogeosciences Discussions, submitted* (2014).

[2] Horsburgh, J. M., Morrice, M., Lea, M.-a. & Hindell, M. A. Determining feeding events and prey encounter rates in a southern elephant seal: a method using swim speed and stomach temperature. *Marine mammal science* **24**, 207–217 (2008).

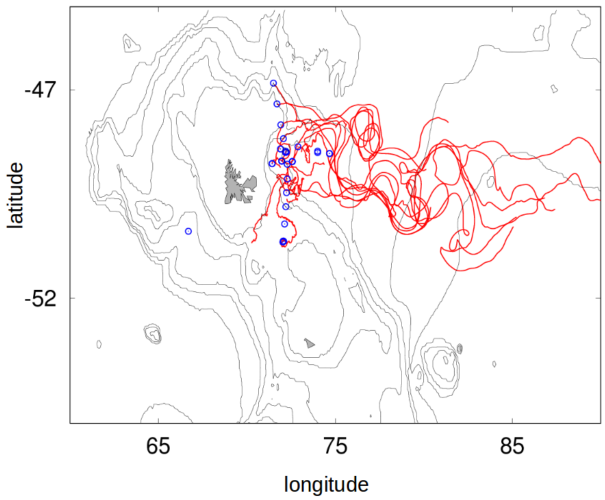

Figure 1: Distribution of in-situ physics measurements used in this study. The blue dots represent the location of the ADCP casts and the red lines the trajectories of some of the drifting buoys.

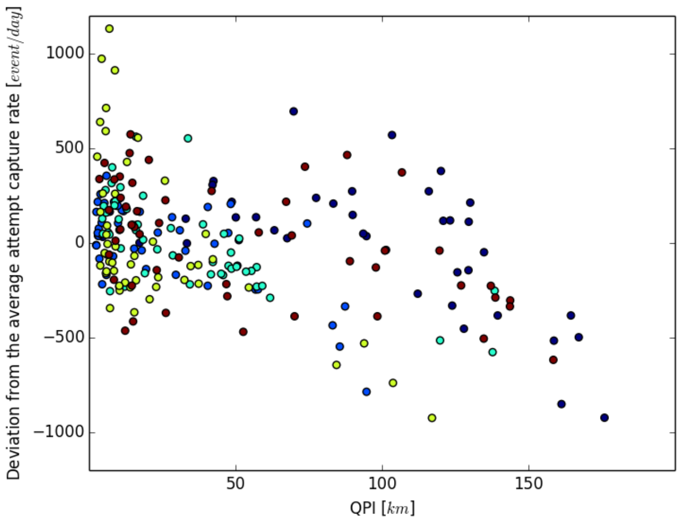

Figure 2: The QPI and deviation from the individually-averaged capture rates. Different colors refer to different individuals.

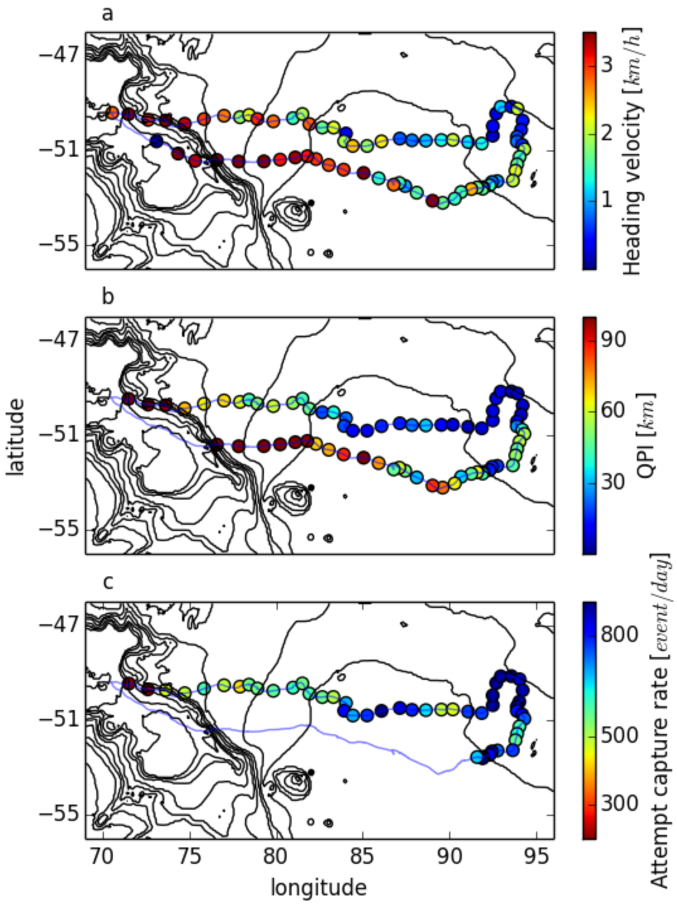

Figure 3: Example of computation of heading velocity (a), QPI (b) and attempt capture rate measurements (c) along an elephant seal's trajectory. Note that the colorscale in (c) is reversed. The patterns along this and the other trajectories of the attempt capture rate, heading velocity and the QPI include, in agreement with previous observations [2] of their foraging habit, an inbound and an outbound phase of the trajectory, with a lower attempt capture rate and high QPI, with an intensive foraging and low QPI phase in between.

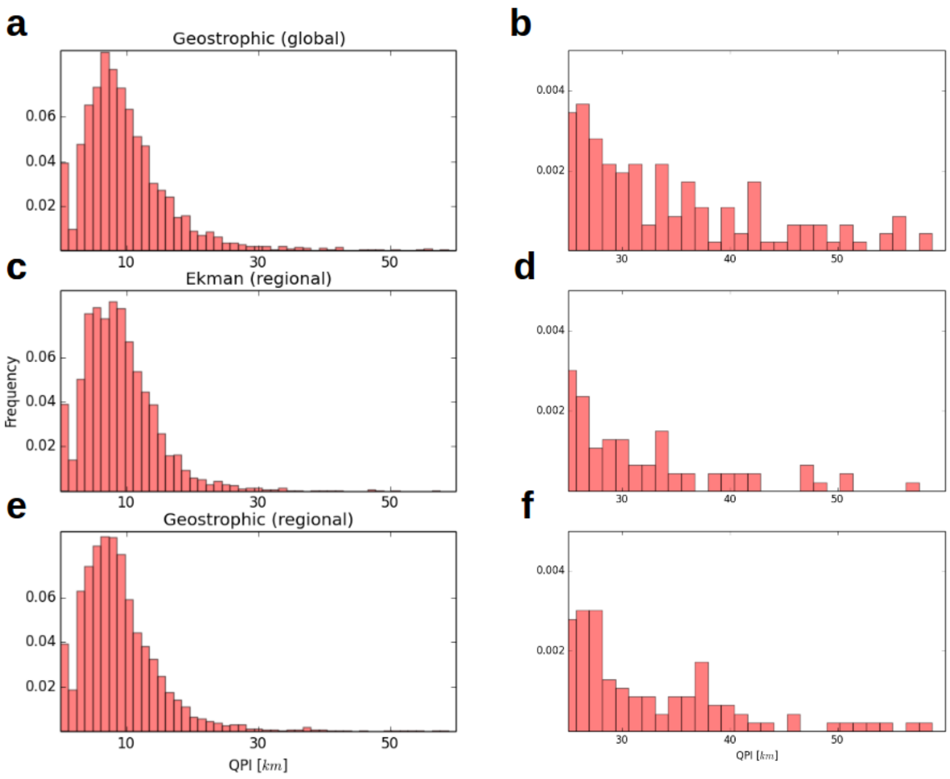

Figure 4: Distributions of the QPI for SVP (real) drifters computed using different altimetry products: a) geostrophic global product, c) Ekman-corrected regional product and e) geostrophic regional product. Using different products does not alter significantly the shape and the extent of the distribution, yet differences in the distributions can be observed in the tails, as displayed in b), d) and f).
